# Modified Uterine Allotransplantation and Immunosuppression Procedure in the Sheep Model

**DOI:** 10.1371/journal.pone.0081300

**Published:** 2013-11-22

**Authors:** Li Wei, Tao Xue, Hong Yang, Guang-Yue Zhao, Geng Zhang, Zhi-Hong Lu, Yan-Hong Huang, Xiang-Dong Ma, Hai-Xia Liu, Sheng-Ru Liang, Fang Yang, Bi-Liang Chen

**Affiliations:** 1 Department of Obstetrics and Gynecology, Xijing Hospital, The Fourth Military Medical University, Xi’an, People’s Republic of China; 2 Department of Otorhinolaryngology, Xijing Hospital, The Fourth Military Medical University, Xi’an, People’s Republic of China; 3 Department of Osteology, Xijing Hospital, The Fourth Military Medical University, Xi’an, People’s Republic of China; 4 Department of Urinary Surgery, Xijing Hospital, The Fourth Military Medical University, Xi’an, People’s Republic of China; 5 Department of Anesthesiology, Xijing Hospital, The Fourth Military Medical University, Xi’an, People’s Republic of China; Faculty of Animal Sciences and Food Engineering, University of São Paulo, Pirassununga, SP, Brazil, Brazil

## Abstract

**Objective:**

To develop an orthotopic, allogeneic, uterine transplantation technique and an effective immunosuppressive protocol in the sheep model.

**Methods:**

In this pilot study, 10 sexually mature ewes were subjected to laparotomy and total abdominal hysterectomy with oophorectomy to procure uterus allografts. The cold ischemic time was 60 min. End-to-end vascular anastomosis was performed using continuous, non-interlocking sutures. Complete tissue reperfusion was achieved in all animals within 30 s after the vascular re-anastomosis, without any evidence of arterial or venous thrombosis. The immunosuppressive protocol consisted of tacrolimus, mycophenolate mofetil and methylprednisolone tablets. Graft viability was assessed by transrectal ultrasonography and second-look laparotomy at 2 and 4 weeks, respectively.

**Results:**

Viable uterine tissue and vascular patency were observed on transrectal ultrasonography and second-look laparotomy. Histological analysis of the graft tissue (performed in one ewe) revealed normal tissue architecture with a very subtle inflammatory reaction but no edema or stasis.

**Conclusion:**

We have developed a modified procedure that allowed us to successfully perform orthotopic, allogeneic, uterine transplantation in sheep, whose uterine and vascular anatomy (apart from the bicornuate uterus) is similar to the human anatomy, making the ovine model excellent for human uterine transplant research.

## Introduction

Uterine-factor infertility includes cases of absence of the uterus or those with nonfunctional uterus in terms of pregnancy capability and occurs due to congenital or acquired causes such as uterine agenesis, hysterectomy, uterine hypoplasia, arcuate uterus and intrauterine adhesions [1]. The overall prevalence of uterine-factor infertility is approximately 3%–5% in the general population, and the preservation of fertility in these patients is challenging. Most women with uterine-factor infertility cannot become genetic mothers, except through the use of gestational surrogacy. Surrogacy and adoption are temporary solutions for uterine-factor infertility. Moreover, whether these services are valid options for reproduction or simply brokered commodities is much debated. Many argue that these techniques do not adequately meet the needs of infertile couples who wish to carry their own biological child to term [2]. The logical but radical treatment approach would be uterine transplantation, which could allow these women to become both genetic and gestational mothers [3].

Advances in immunosuppression have decreased the risk of rejection and expanded the transplantation field, which was restricted to vital (heart, liver, lung) or near-vital (kidney) organs, to include organs whose absence or malfunctioning interferes with the fulfillment of a normal life, such as the hand [4,5], larynx [6,7], forearm [8], thumb, face [9] and small bowel [10]. Uterine transplantation is a proposed, quality-of-life, transplant procedure to cure permanent uterine-factor infertility.

Uterine transplantation has been researched in several animal models, including mice [11,12], rats [13], sheep [14–16], pigs [17], baboons [18] and macaques [19,20]. In 2000, the first human uterine transplantation was performed in Saudi Arabia; however, the graft was removed after 99 days due to failed reperfusion of the vasculature [21]. Some experts attributed this failure to the limited animal research conducted by the surgical team. To address the concerns regarding the risks of this procedure, researchers must develop different uterine transplantation models. These models can then be used to study methodology including organ recovery, ischemia-reperfusion and transplantation surgery, also to establish ways of inducing tolerability to an allogeneic transplanted uterus, harboring a pregnancy to term in the transplanted uterus and delivering live offspring [1]. Thus, regardless of how substantial the bodies of animal work, all studies raise more questions and call for more animal studies. Animal models are currently being reported by multiple groups from around the world.

This pilot study aimed to develop and evaluate a technique for orthotopic, allogeneic uterine transplantation in sheep, and provide information about immunosuppression procedures to assist future experimental studies.

## Subjects and Methods

### Animals

Ten mature female sheep were purchased from an accredited sheep reproductive biotechnology center, where each animal was identified as being unrelated and fertile. The selection criteria were a previous pregnancy, age of 2–3 years, weight between 35 and 50 kg and absence of parasitic infection. ABO and human leucocyte antigen (HLA) tissue matching were not performed because large subprimate animals have numerous HLAs [15]. The animals arrived at the surgical facility 2 weeks before the experiments commenced and were kept in indoor shelters, with access to the outdoors during daylight hours. The shelters were maintained at 20°C and had a controlled 12 h/12 h light-dark cycle. The animals had free access to hay and water. To allow drainage of the rumen at the time of surgery, hay was withdrawn 48 h before surgery to minimize the fiber content in the rumen. Our research protocol was approved by the Animal Care and Use Committee of the Fourth Military Medical University, P. R. China.

All animals were placed under general anesthesia with the standard veterinary protocol. Two operations were performed simultaneously where each animal served as a donor as well as a recipient. A control group of sheeps with nontransplanted uteruses were used to compare uterine histology and blood samples with the transplant recipients. 

### Anesthesia protocol

On the day of the experiment, the animals were brought to the operating room and intramuscularly injected with ketamine (20–22 mg/kg) and 2% xylazine hydrochloride (0.2–0.4 mg/kg) to induce anesthesia [22]. General anesthesia was maintained using a continuous intravenous propofol infusion (10 mg/ml) at a rate of 25 ml/h. The ewe was then positioned in ventral recumbency.

Heart rate, respiratory rate and mean arterial pressure were measured before anesthesia, at 5-min intervals after the induction of anesthesia for up to 30 min and during recovery. In all sheep, the durations of anesthesia induction, anesthesia and recovery were recorded. The quality of induction, anesthesia, analgesia and recovery were also evaluated.

To compensate for fluid loss during the experimental procedure, each animal received a continuous intravenous infusion of voluven (4–5 ml/kg/h) and Ringer’s lactate solution (5–6 ml/kg/h). Glucose solution (10%) was administered when blood glucose levels were below 3.5 mmol/l.

### Surgical procedure for graft procurement

The sheep was positioned in a supine position. The wool on the lower abdomen was clipped, and the region was disinfected with povidone iodine solution and draped. All surgical procedures were performed using sterile techniques. The urinary bladder was catheterized through the urethra with a balloon catheter (outer diameter, 4.7 mm). Using a minimally invasive approach, we operated on two sheep simultaneously, with each ewe serving as the donor for the other A 7-cm vertical midline incision was made; during this stage, care was taken to avoid the large subcutaneous mammary vein in the midline of the abdomen. The rumen, small bowel and major part of the colon were positioned up into the cranial portion of the abdomen by manipulation and held there with three abdominal retracting pads (450 mm). A self-retaining abdominal retractor was used during the subsequent surgery to expose the underlying pelvic viscera and retroperitoneal structures.

**Figure 1:**
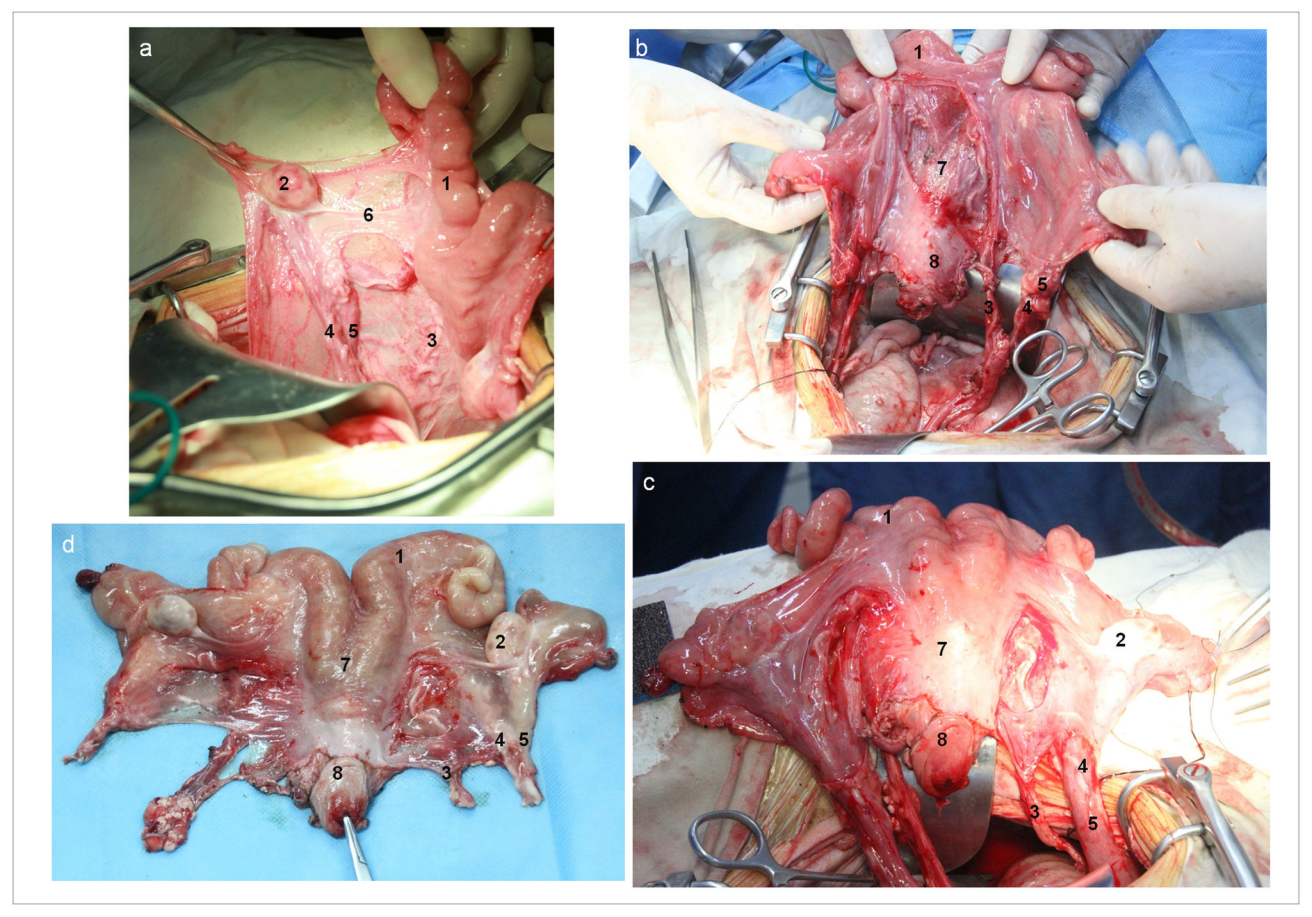
Uterine anatomy and vasculature at various stages of the graft-procurement procedure. (a) Three abdominal retracting pads provided excellent exposure for microdissection of the sheep uterus, adnexa and their vasculature. (b and c) The uterus and vessels before excision from the posterior surface (b) and the ventral surface(c). (d) The excised uterus before transplantation. (Hysterectomy was started with division of the round ligaments using a cautery. The broad ligament was opened, both the uterine arteries/ veins and both the utero-ovarian arteries/ veins along with both the ureters were carefully dissected from the peritoneal surface. The vessels were dissected free down to a level 20 mm distal to their branching from the internal iliac vessels. The large veins and arteries of the vagina and cervix were ligated and severed at the level of the planned transection of the vagina. The vagina was then divided approximately 5 mm caudal to the cervix. Eight atraumatic vascular clamps were applied to each uterine and utero-ovarian artery and vein, securing the proximal and distal ends of the uterine vasculature. Heparin was then administered. Finally, all the vessels were transected using microsurgical scissors, and total abdominal hysterectomy with oophorosalpingectomy was achieved.). 1, Left uterine horn; 2, left ovary; 3, left uterine artery; 4, left utero-ovarian artery; 5, left utero-ovarian vein; 6, suspensory ligament of left ovary; 7, corpus uteri; 8, cervix uteri.

The aim of the surgery was to isolate a specimen that included the common uterine cavity, cervix, both uterine horns, both ovaries and oviducts, the upper vagina and a vascular pedicle, including the right and left uterine and utero-ovarian arteries and veins. The first procedure, after obtaining full pelvic access, was to evaluate the uterus and ovaries for structural abnormalities. Then, the right and left utero-ovarian ligaments, and uterine and ovarian vessels were identified (Figure 1b,c). The uterus receives most of its blood supply from the two uterine arteries and two utero-ovarian arteries. We harvested both the uterine arteries and both the utero-ovarian arteries up to a point approximately 2 cm distal to their origins from the anterior branch of the internal iliac artery, and used the dissected vessels for subsequent arterial anastomosis. The same selection was made for uterine and utero-ovarian veins for venous anastomosis. In some animals, the uterine veins were difficult to identify, and we chose only the utero-ovarian veins for venous anastomosis.

Hysterectomy was started with division of the round ligaments using a cautery. The broad ligament was opened, and the ureters identified. All the vessels mentioned above along with both the ureters were carefully dissected from the peritoneal surface of the broad ligament. Subsequently, the vessels were dissected free down to a level 20 mm distal to their branching from the internal iliac vessels. During the dissection procedure, unipolar diathermy (15–40 W; Valleylab Bipola) was used to minimize bleeding. After this step, the uterus was pulled cranially by gentle traction to carefully separate the bladder and rectum from the uterus and upper vagina. The large veins and arteries in the midline and at the lateral aspects of the vagina and cervix were doubly ligated and severed at the level of the planned transection of the vagina. The vagina was then divided by unipolar diathermy, approximately 5 mm caudal to the cervix (Figure 1d). Eight atraumatic vascular clamps were applied to each uterine and utero-ovarian artery and vein, securing the proximal and distal ends of the uterine vasculature. Heparin (12,500 U) was then administered intravenously. Subsequently, both the right and left vessels were transected using microsurgical scissors, and total abdominal hysterectomy with oophorosalpingectomy was achieved.

### Graft preparation

The uterus was transferred and preserved in a sterile basin, and then chilled in ice at 4°C. Using a 23-gauge intravenous catheter, we irrigated the uterus with histidine-tryptophan-ketoglutarate buffering solution (Custodiol HTK, Shanghai, China) at the level of both the uterine and utero-ovarian arteries. Sodium heparin (12,500 U) and xylocaine (100 mg) were diluted in HTK solution (1 liter) to flush any residual blood from all the uterine vessels. Continuous perfusion pressure was maintained at a flow of 75 mm Hg. When a total cold ischemic time of 60 min was achieved, each donor uterus was transplanted in the recipients.

### Surgical procedure for transplanting prepared grafts

All vascular uterine pedicles (six to eight) were identified (two uterine arteries, two utero-ovarian arteries and two utero-ovarian veins, with/without two uterine veins), and the donor uterus was positioned anatomically into the pelvic cavity. The donor and recipient vaginal tissue were approximated with 2-0 Vicryl sutures (polyglactin 910; Ethicon) in a continuous, non-interlocking fashion to avoid tissue ischemia. Magnifying loops 2.5X (Leica M525; Leica Microsystems AG, Singapore) were required for vascular anastomosis.

### End-to-end vascular anastomosis

To perform vascular anastomosis, the veins were first approximated with 7-0 polypropylene sutures (Prolene 318-13 mm, Ethicon; Figure 1), and the clamps on the veins were released to evaluate venous patency. After documenting venous patency, the donor and recipient uterine arteries were approximated with 8-0 polypropylene sutures; again, the vascular clamps were released, and adequate reperfusion of the donor uterus was demonstrated. All uterine vessels were approximated using the end-to-end anastomosis technique with continuous, non-interlocking sutures to avoid ureter immobilization and fistula formation.

After the vascular anastomosis was completed, interrupted 3-0 Vicryl sutures (polyglactin 910; Ethicon) were used to attach the graft to the peritoneum on the pelvic side, to the native round ligaments and to the uterosacral ligaments, in order to immobilize the uterus and avoid poor fixation of the graft. Poor graft fixation was the reason the first published human uterine transplantation attempt failed, as it led to uterine prolapse and necrosis [21].

The abdominal incision was closed with continuous sutures (Polysorb 1, Covidien), and the skin was closed with interrupted sutures (5-0 polypropylene). The sheep were kept for 24 h in a closed room adjoining the operating room and then transferred to their regular holding space.

We collected the following data during transplantation: transplant procurement time, duration of cold ischemia (time the graft spent in the ice basin), duration of first phase of warm ischemia (time between applying the arterial clamp and chilling in the ice basin), duration of the second phase of warm ischemia (time between removal of the graft from the ice basin and removal of the arterial clamp), total duration of warm ischemia and overall surgery time, including transplant retrieval and transplantation. All these parameters were expressed in terms of the median and range.

### Immune suppression

For immunosuppression, we selected modern induction therapy with antithymocyte globulin (Imtix-Sangstat, France) to lower the number of circulating T cells, followed by standard triple immunosuppression (tacrolimus, corticosteroids and mycophenolate mofetil [MMF]). The immune suppression protocol was initiated 2 d before transplantation and consisted of tacrolimus (Astellas Pharma, Fujisawa Ireland Ltd.; 4 mg/d, i.e., 0.02–0.15 mg/kg/d, orally) and MMF (Roche, Shanghai, China; 1.5 g/d orally). In addition, antithymocyte globulin (50 mg) was administered intravenously for at least 4 h, beginning from the start of the uterus transplantation operation. Corticosteroid treatment consisted of methylprednisolone (500 mg) administered as a single intravenous injection during the surgery (before transplantation of the uterus graft), followed by a daily dose of 40 mg methylprednisolone (Pfizer Italia Srl, Ascoli Piceno, Italy) orally on postoperative days 1–15. From the third week, methylprednisolone was tapered down to a daily dose of 16 mg orally, and then maintained at this level.

For each sheep, serum tacrolimus levels were recorded every third day until a serum trough level of 3–6 mg/l was reached. The animals were followed up for 2–3 months in preparation for the embryo-transfer procedure. A maintenance dose of 6 mg/d tacrolimus was used, and adjustments were made when pregnancy was confirmed.

All the serum levels were measured in the laboratory of the Department of Pharmacology and Toxicology in the University Hospital of the Fourth Military Medical University, P. R. China.

### Postoperative care and follow-up

All animals were managed under sterile precautions and kept in kennels for approximately 5 d. Vital signs, food and water intake, vaginal discharge and rectal temperatures were recorded daily. Moxifloxacin hydrochloride (400 mg/d orally; Bayer AG) was administered for 7 d as preventive antibiotic therapy. Serum chemistry profiles and blood counts were recorded every week for 2 months to detect infections or complications related to the treatment because of the potential hematologic and renal toxicity.

Animals recovered well and were followed up by a veterinary resident and an active faculty member of the Fourth Military Medical University, P.R. China.

### Assessment of graft viability

During the operation, blood was taken from the jugular vein (peripheral sample) and the utero-ovarian vein. Blood samples were collected when the uterus was in situ before the occlusion of the arterial flow and at various time points during reperfusion，which occurred between the period of cold ischemia outside the body and the period of warm ischemia when the vascular anastomoses were established. Blood samples were immediately analyzed for pO_2_, pCO_2_, pH and lactate by a blood gas analyzer (GEM Premier 3000).

After the surgery, serial transrectal ultrasonography scans (to avoid vaginal contamination; Voluson E6, GE Healthcare, USA) were obtained at every week from 2 weeks to 2 months after the transplantation procedure, in order to evaluate acute rejection and uterine structure. To inspect the graft and the anastomosis site, a “second-look” median laparotomy was performed 4 weeks after the transplantation procedure. During this second operation, the graft was removed, if it did not appear viable, and examined for signs of rejection or necrosis.

### Histology

A total abdominal hysterectomy with oophorosalpingectomy was performed in one sheep for histologic studies. Biopsy specimens from the uterus, cervix, ovary, fallopian tube, liver and kidney were embedded in paraffin, sectioned and stained with hematoxylin-eosin. All the sections were analyzed by light microscopy for signs of ischemic injury, infection, thrombosis and immunological rejection. Thus, edema, stasis, necrosis, apoptosis and neutrophil count were noted in each section.

### Statistical analysis

The results of blood analyses were expressed as medians and individual values. The nonparametric, Wilcoxon signed-rank test was used to compare the first value (10 min) with the 60-min value. A P value of <0.05 was considered significant.

## Results

### Surgical outcomes

After the first two transplantation attempts in March 2012, some modifications were introduced since these initial experiments were unsuccessful. One death occurred owing to postoperative enterogastric reflux and aspiration at 1 h after extubation of the tracheal tube. We therefore modified the anesthesia protocol to intramuscular injection of ketamine and xylazine hydrochloride [22].

**Figure 2:**
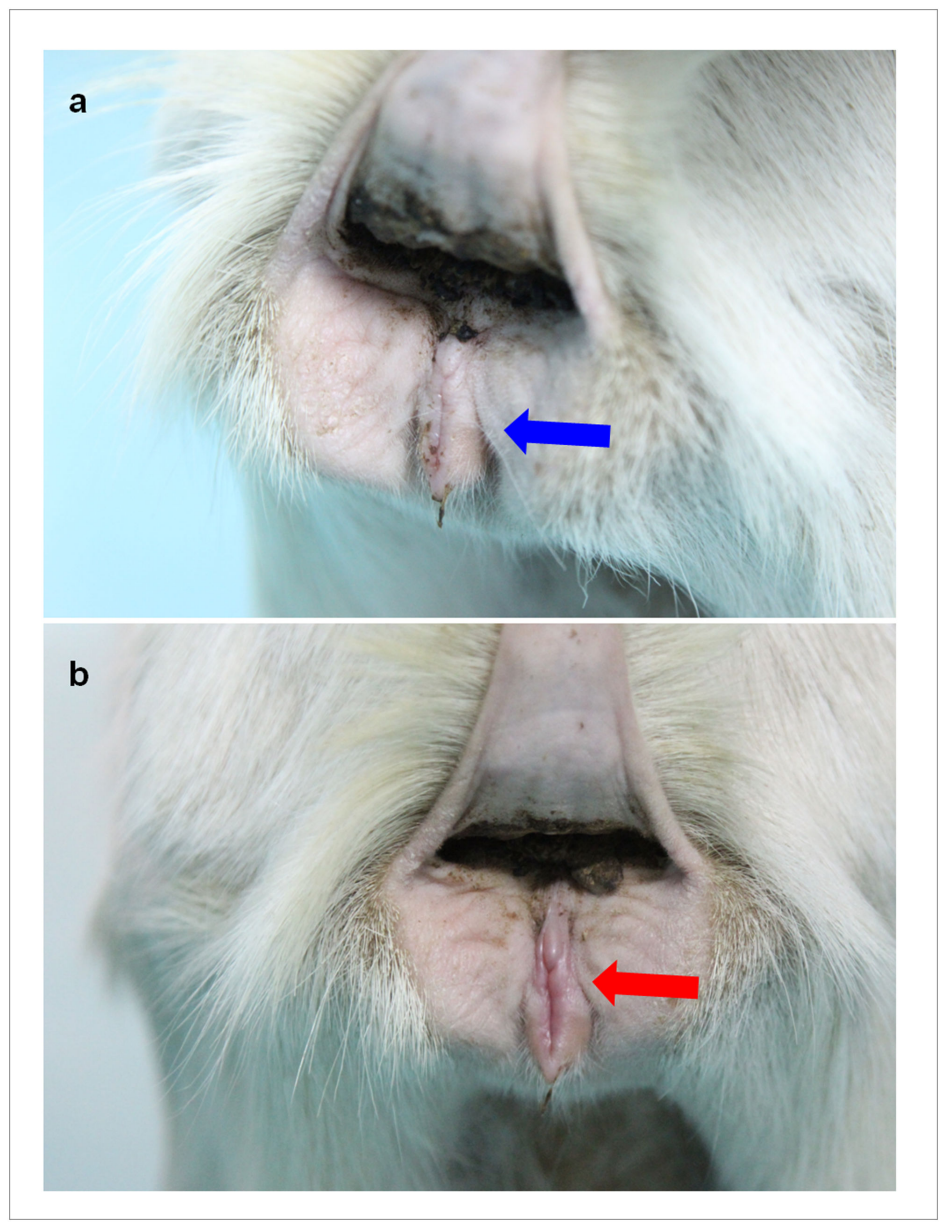
Changes in the vulvar skin due to resumption of ovarian cyclicity in the transplanted sheep. (a) Appearance of the vulvar skin when the ewe is not in estrus. (b) At 92 days after uterine transplantation, the vulvar skin of ewes 5 showed congestion, swelling and relaxation; which were signs of estrus. The same changes were observed on ewes 8 at 118 days after the transplantation. Thus, vulvar skin changes could be used as a non-invasive measure of successful transplantation.

We then carried out eight surgeries between March and September 2012. The median duration of these surgeries (retrieval and transplantation) was 395 min (260–400 min). The median duration of transplant procurement was 155 min (130–230 min). The median time of warm ischemia was 10 min (8–15 min). The median time of cold ischemia was 170 min (130–270 min). Of the 10 sheep, ewe 1 died of postoperative enterogastric reflux and aspiration. Ewe 3 suffered perioperative bleeding and died 13 h after the operation. Ewe 4 developed diarrhea, followed by severe vaginal and rectal infection 3 months after the transplantation; an exploratory laparotomy was performed to evaluate the status of the transplanted uterus in this sheep. Of the remaining seven uterine allograft recipients, none developed postoperative peritonitis or uterine prolapse; therefore, these seven sheep were used as potential candidates for natural conception or embryo transfer. Of these sheep, ewes 5 and 8 showed signs of estrus at 92 and 118 days after the transplantation, respectively (Figure 2).

### Intraoperative graft evaluation

**Figure 3:**
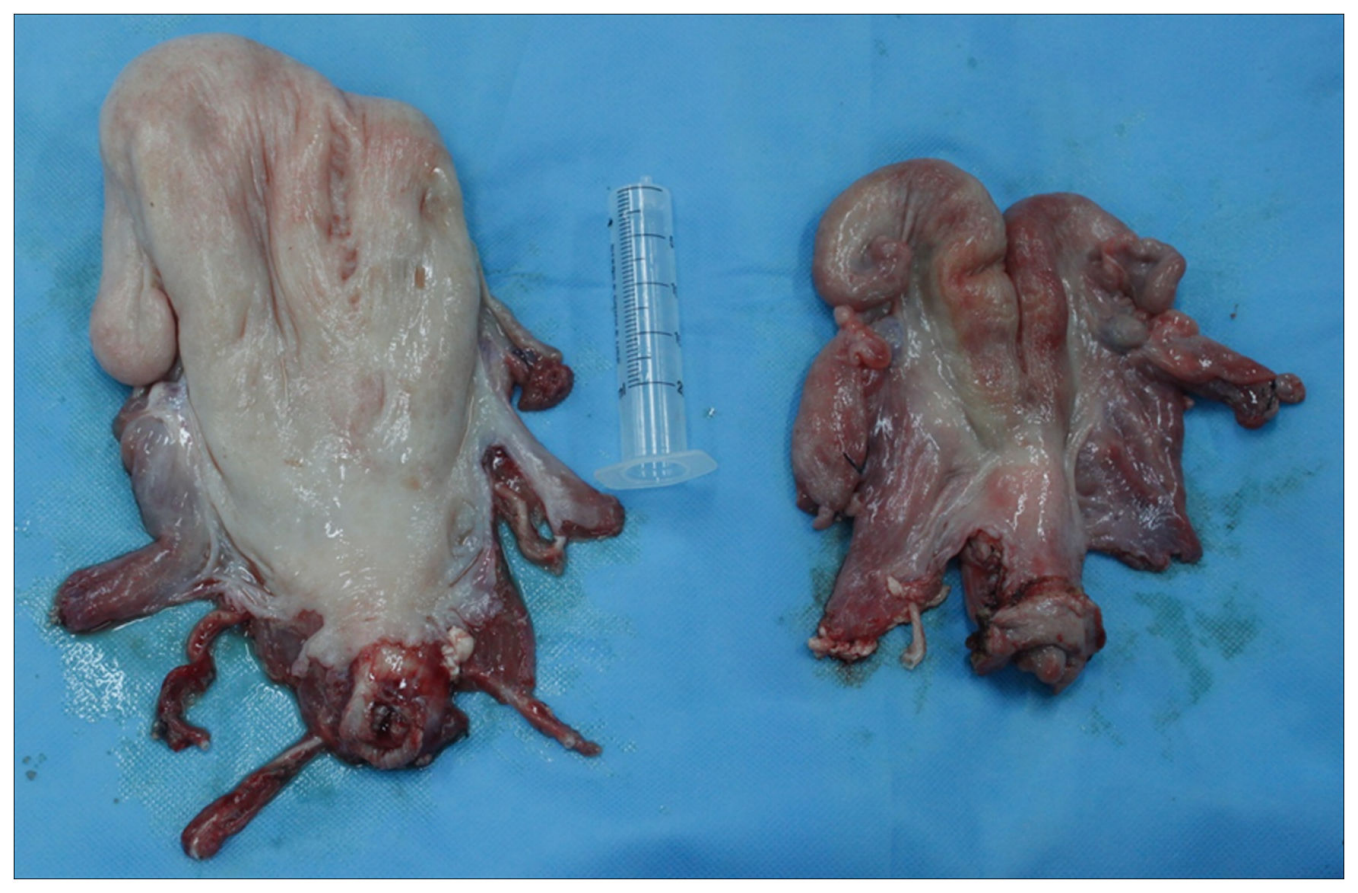
The excised uterus was transferred and preserved in a sterile basin, and then chilled in ice at 4°C. **Comparing with uterus in vivo, after perfusion of HTK renal preservation solution through the uterine arterial system, clear blanching of the uterine tissue was observed**. Significant uterine size difference when the uterine allotransplantation were carried out between a normal ewe (ewe 5) and a puerperous ewe which delivered two days before (ewe 6). (a) Postpartum uterus of ewe 6; (b) normal uterus of ewe 5.

**Figure 4:**
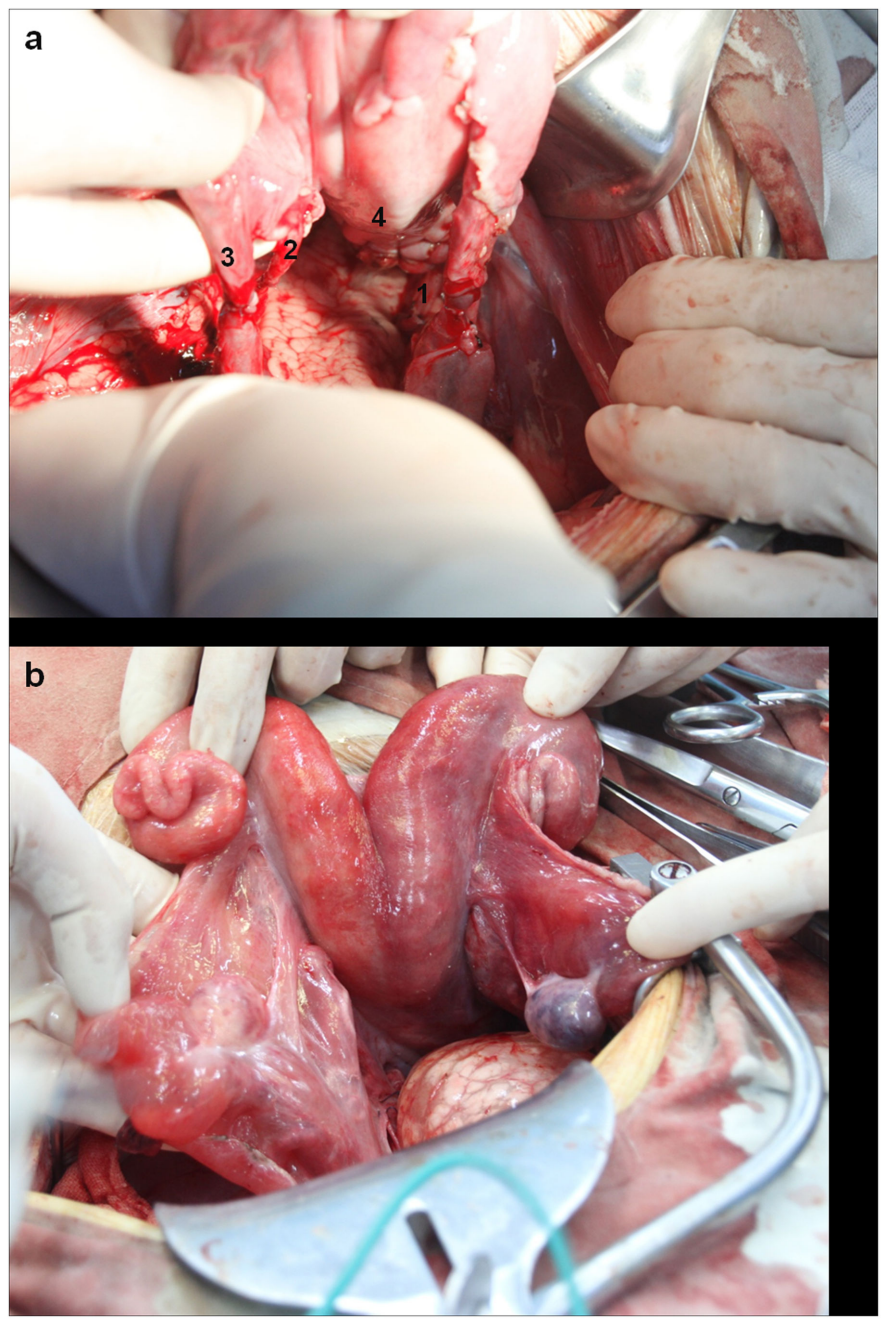
Bicornuate transplanted uterus and sites of vascular anastomosis. (a) Sites of utero-vaginal anastomosis and end-to-end vascular anastomoses. 1, Anastomosis site of left uterine artery; 2, anastomosis site of right utero-ovarian artery; 3, anastomosis site of right utero-ovarian vein; 4, anastomosis site of vagina. (b) Comparing with Figure 3, after re-initiation of blood flow, the color of the uterus rapidly changed from pale to reddish.

In all allografts, we observed clear blanching of the uterine tissue (Figure 3) after in situ perfusion of HTK renal preservation solution through the uterine arterial system. In all transplanted ewes, recirculation in the graft was satisfactory, as determined using graft staining. We performed end-to-end anastomosis of the vessels (Figure 4a). After the vessels around the sites of vascular anastomoses were unclamped to reestablish the blood flow, the uterine color rapidly (within 1–2 min) changed from pale to reddish (Figure 4b) in all sheep. We also confirmed the satisfactory recirculation in the graft by noting substantial venous blood flow after puncture of the utero-ovarian vein after clamp removal.

### Blood parameters

Blood samples were collected every 30 min throughout the ischemic period, beginning from the application of the atraumatic vascular clamps and transection of the vessels to the vascular anastomosis and clamp removal. Among which, the samples collected at the end of warm ischemia (median duration, 10 min) were also tested.

**Figure 5:**
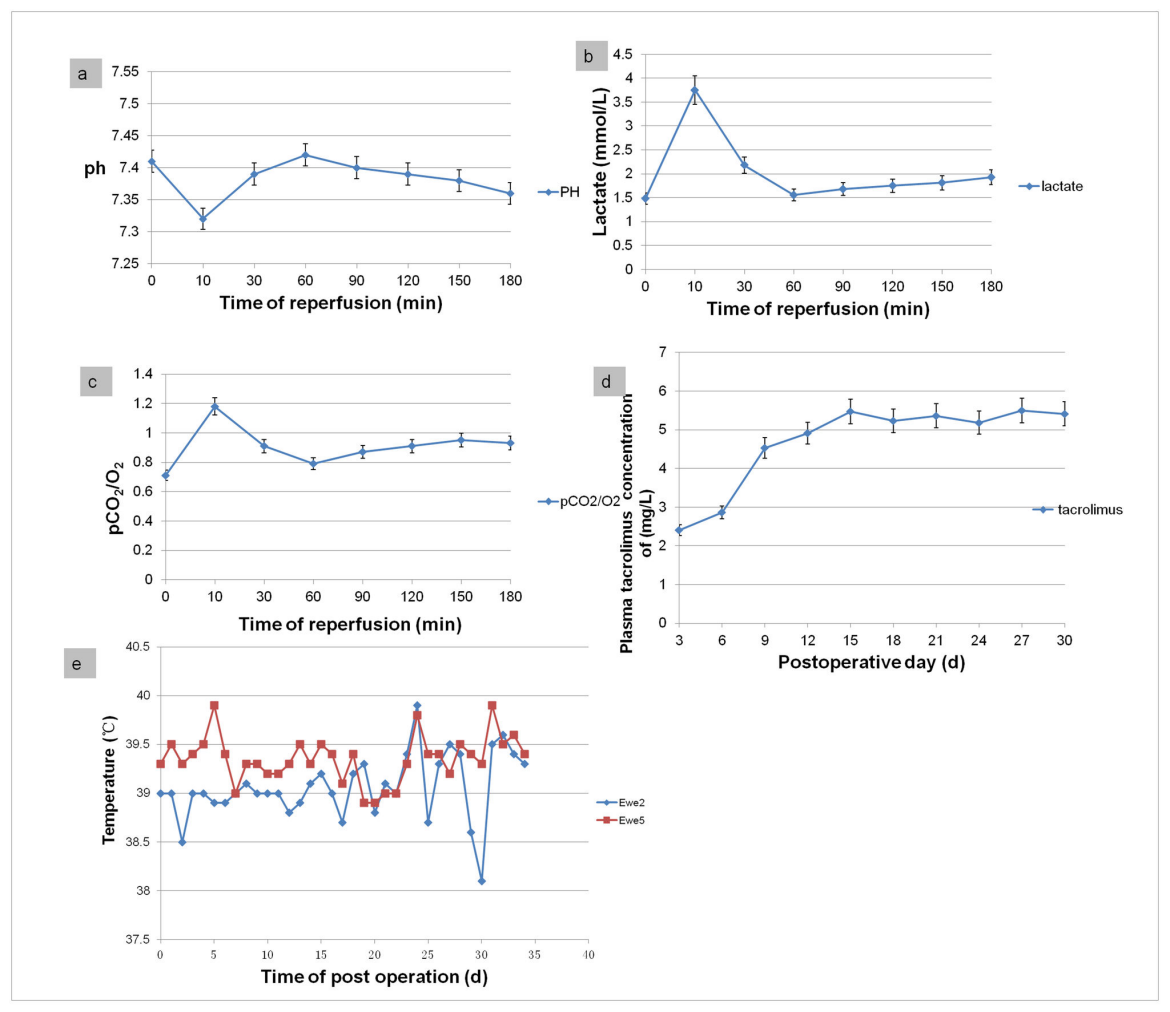
Blood gases and metabolite concentrations at different time points in venous blood samples obtained from the transplanted uterus during reperfusion. (a), pH levels; (b), lactate concentrations; (c) ratio between pCO_2_ and pO_2_ were achieved from the transplanted sheep during the 180 min of reperfusion. (d) Appropriate tacrolimus trough levels were achieved 15 days after uterin transplantation. (e) Smooth postoperative temperature curve indicated reasonable immunosuppression and infection prevention.

The pH of the venous effluent of the transplanted uterus considerably decreased at 10 min compared to the baseline (0 min) value; it then increased steadily from 10 min to 60 min during reperfusion and gradually decreased thereafter (Figure 5a). The pCO_2_/pO_2_ ratio significantly increased at the end of warm ischemia, during cold ischemia at 4°C and perfusion of the HTK renal preservation solution, it decreased steadily until 60 min of reperfusion (Figure 5c). The lactate levels also initially increased and were significantly higher at 10 min; during cold ischemia at 4°C and perfusion of the HTK renal preservation solution, it also descend gradually (Figure 5b).

### Immune suppression

 Among the seven surviving sheep, the surgeries were well tolerated. The serum tacrolimus levels in the sheep were recorded on every third day and showed great interindividual variability. The median area under the curve (AUC) of tacrolimus in the first postoperative week was 2.63 mg/l (1.33–4.54 mg/l). We then switched the sheep to oral tacrolimus (4–6 mg/d), and the median AUC of tacrolimus increased to 5.32 mg/l (3.57–7.91 mg/l; Figure 5d).

### Follow-up

On postoperative day 2, the white blood cell count of the transplanted sheep was 12,000–25,000/l. These elevated counts were managed with moxifloxacin hydrochloride 400 mg/d orally for 7 d. The median rectal temperature during the first 30 d after transplantation was 39.2°C (38.1–39.9°C). There was no fever. The food and water intake was the same as that before transplantation (Figure 5e).

During the first 9 postoperative days, ewes 2, 4 and 6 occasionally discharged scant (approximately 2–4 ml), dark red vaginal secretions, which had no fetid smell. None of the other sheep had any vaginal discharge.

### Postoperative graft assessment

Serial transrectal ultrasonography was performed at 2 weeks after the surgery to evaluate acute rejection and uterine structures. In all transplanted ewes, the uterus with both horns and the ovaries were identified, and the myometrial and endometrial signal intensity was similar to that in the controls. We observed no necrosis or edema. Doppler ultrasonography showed no stasis in any of the vascular anastomoses, indicating that satisfactory recirculation was established in the graft in all eight ewes.

**Figure 6:**
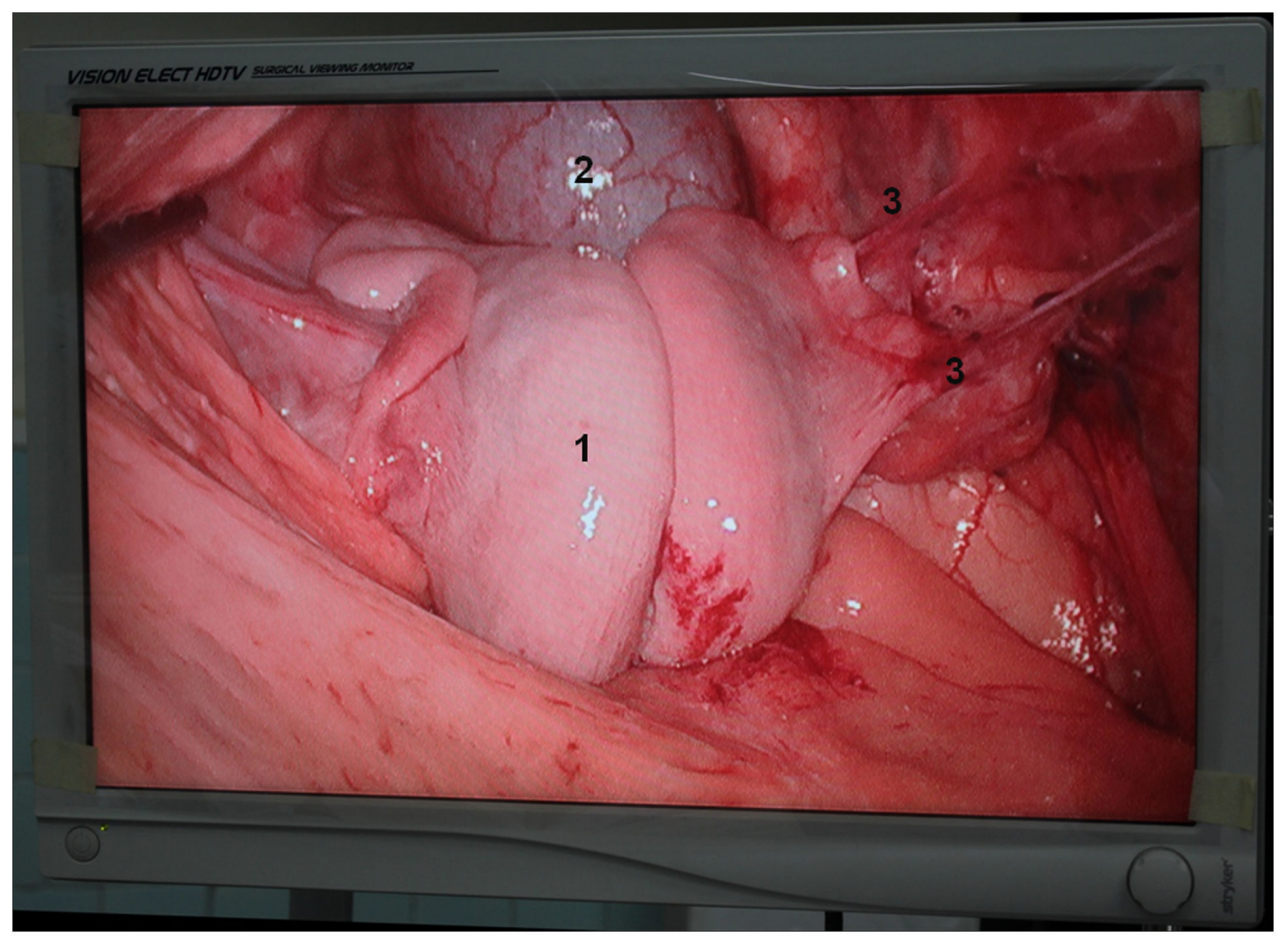
Exploratory laparoscopy performed 1 month after the transplantation procedure. Macroscopically, normal-sized uterus with normal-sized ovaries were seen in the transplantated sheep. 1, transplanted uterus; 2, bladder; 3, Adhesions between the uterus and anterior peritoneum.

One month after the transplantation procedure, exploratory laparoscopy was performed in two of the transplanted sheep (ewes 2 and 7) to evaluate the status of uterine tissue structure and vascular patency. Abdominal adhesions were observed in both animals. Ewe 2 had omental adhesions to the anterior abdominal wall wound. In both ewes, meticulous sharp dissection was performed to prevent injury to the adjacent organs. The pelvic cavity was then examined to further evaluate the outcome of the transplanted allograft. Macroscopically, normal-sized uteruses with normal-sized ovaries were seen in both sheep (Figure 6). Adequate blood flow to the uterine tissue was verified by observing substantial blood flow after incision of the myometrium.

### Histology

At 3 months after transplantation, ewe 4 developed diarrhea, followed by a severe vaginal and rectal infection. An exploratory laparotomy was performed to evaluate the status of the uterine tissue. On gross inspection, the two horns of the uterus were united caudally, and the site of the uterine-vaginal anastomosis was intact. In the pelvic cavity, the uterus was a normal reddish color, and no signs of vascular thrombosis were found. Substantial blood flow occurred upon incision of the myometrium, which confirmed an adequate uterine blood flow. However, the bladder and stomach were dilated and appeared purple-to-black because of necrosis. Therefore, a total abdominal hysterectomy with oophorosalpingectomy was performed for histologic studies. Additionally, liver and renal resections were carried out for histologic studies to evaluate the influence of immune suppression.

**Figure 7:**
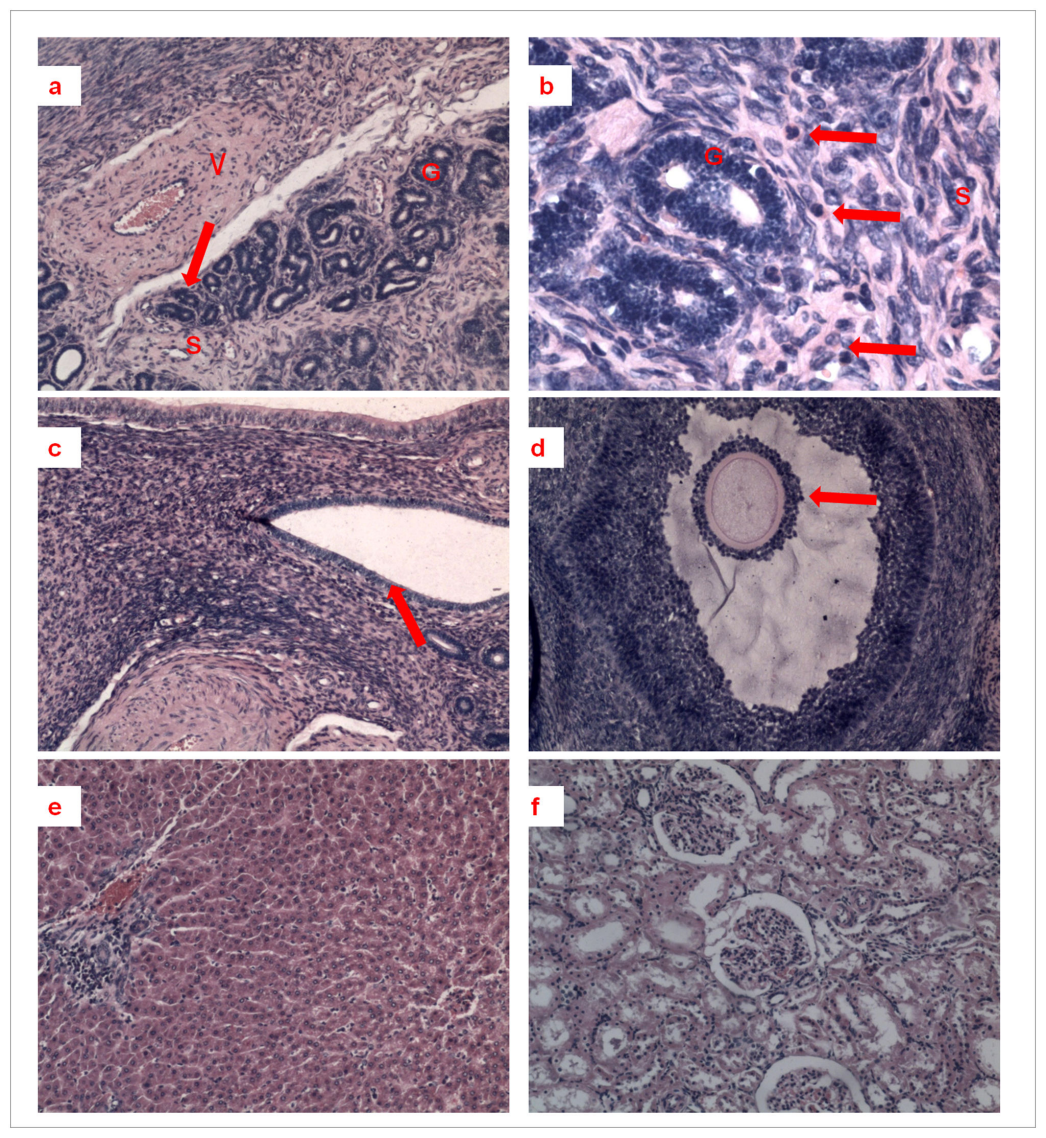
Histological examination of various organs after uterine allotransplantation. Light micrographs of uterine tissue (a and b). S, stroma; G, endometrial gland; V, blood vessel. Hematoxylin–eosin stain; magnification, (a) 100× and (b) 400×. The area indicated by the arrow in (a) is shown at a higher magnification in (b). The arrows in (b) indicate neutrophilic granulocyte infiltration. Light micrographs of (c) cervical and (d) ovarian tissue. Hematoxylin–eosin stain; magnification, 100×. The arrow in (c) shows typical cervical columnar epithelium. The arrow in (d) shows a typical secondary follicle. Light micrographs of (e) hepatic and (f) renal tissue. Hematoxylin–eosin stain; magnification, 100×. Both specimens have a normal appearance with few infiltrating neutrophils.

Compared with the control group, the transplanted uterus showed endometrial tissue, abundant smooth muscle and neutrophilic infiltration of the endometrium (Figure 7a,b). Thus, a typical structure with a very subtle inflammatory reaction was found in the transplanted uterus. Moreover, the transplanted cervix and ovaries also showed a normal architecture (Figure 7c,d). We observed no necrosis, edema, apoptopic cell fragments or vascular stasis in any of the samples. The hepatic and renal tissues also appeared normal, with few infiltrating neutrophils (Figure 7e,f).

## Discussion

Before the advent of modern assisted reproductive technologies (ARTs), reproductive organ transplantation was investigated as a potential treatment for ovarian, tubal and uterine infertility [23,24]. Medical science and clinical medicine have made tremendous advances during the last few decades. One milestone was the first report of a live birth after in vitro fertilization (IVF) [25]. The introduction of IVF during the 1980s expanded the new clinical field of reproductive medicine. New techniques in reproductive medicine have provided a solution to most types of infertility. However, despite this remarkable progress, the management of uterine-factor infertility, which affects millions of women with congenital or acquired uterine conditions worldwide, remains to be resolved.

Uterine transplantation has been on the periphery of medical research for decades [23]. In 2000, the first human uterine transplantation was performed, with the transplantation of the uterus and oviducts in a young woman who had undergone an emergency peripartum hysterectomy [21]. Since then, uterine transplantation has garnered increasing attention as investigators and the public have realized its potential to address absolute uterine-factor infertility [3,26]. Uterine transplantation is now close to becoming a reality through slow, methodical research involving multiple institutions and disciplines. In fact, the recent optimism for its imminent success may be due largely to the breakthroughs in immunosuppressive medicine as well as the development of microsurgery and the emergence of IVF [27]. Successful uterine transplantation will give women with uterine-factor infertility hope, where once there was none, to carry a biological child to term [15].

Most of the available data on uterine transplantation have been derived from animal experiments involving rat, mouse, rabbit, dog, pig, sheep and nonhuman primate models. However, despite the encouraging results, the concept of uterine transplantation raises numerous clinical problems yet to be resolved. Indeed, the best surgical techniques for vascularization of the transplanted uterus as well as the most satisfactory immunosuppressive treatment following transplantation are yet to be established. The purpose of this pilot study was to optimize the surgical technique of uterine transplantation and to provide relevant information about immunosuppression that may assist future experimental studies. We performed orthotopic, allogeneic, uterine transplantation in a sheep model, which was selected to mimic human conditions as closely as possible. The advantage of using sheep is that the body size and pelvic vascular anatomy are similar to those in young women. Moreover, sheep usually carry a singleton or duplex pregnancy, and their gestation lasts only for several months [14].

Uterine transplantation operation involves complicated surgical techniques, ischemic reperfusion injury and ischemic preservation. Selection of the type and number of blood vessels required to maintain uterine blood flow is also important. However, it is unclear how many arteries and veins are required to maintain perfusion of a transplanted uterus. The remarkable complexity of both the pelvic and uterine vascular anatomy accounts for the numerous methods developed to overcome ischemia of the transplanted uterus. The techniques and sites of vascular anastomosis have to be improved because of the high rates of vascular thrombosis. Thus, numerous issues need to be resolved before a safe methodology for human use can be presented. First, we must familiarize ourselves with the physiological vascularization of the uterus. The uterus is supplied with blood mainly by the uterine arteries. The uterine artery originates bilaterally from the anterior division of the hypogastric artery. Upon reaching the side of the uterus, it ascends to the junction of the fallopian tube and uterus, and anastomoses with branches from the ovarian artery, creating an arterial arcade in the upper part of the broad ligament. In addition, descending branches of the uterine artery anastomose with branches from the vaginal artery. Uterine veins parallel the arteries, forming plexuses that drain into the internal iliac vein. The uterine veins merge with the vaginal plexus inferiorly (utero-vaginal venous plexus) and with the ovarian veins superiorly (utero-ovarian plexus) [28]. Thus, the uterus is supplied by six vessels (ovarian, uterine and vaginal). The ample redundant blood supply of the uterus makes the organ suitable for transplantation. On the other hand, the remarkable complexity of the uterine vascular anatomy accounts for the numerous methods developed to overcome ischemia of the transplanted uterus. In abdominal radical trachelectomy, the uterine blood supply has been maintained with only two ovarian vessels in some patients who subsequently became pregnant and delivered a child. In addition, selective ligation of the pelvic vasculature has been used in gynecological procedures that may cause life-threatening hemorrhage from the uterus [29]. Thus, these reports suggest that the ovarian vessels, i.e., only two of the six vessels supplying the uterus, are adequate to maintain uterine viability. The uterine arteries are the main contributors to the blood supply of the uterus. However, it is difficult to determine which veins (ovarian, uterine or deep uterine veins) are the most important [30]. In uterine transplantation experiments, Enskog et al. [16] used the ovarian vein and Kisu et al. [20] utilized the deep uterine vein for uterine perfusion. Indocyanine green (ICG) fluorescence imaging has been used to investigate uterine hemodynamics, and it has shown that blood from the uterine artery is drained mainly by the ovarian veins, rather than the uterine veins [19]. Therefore, we selected six-to-eight uterine vascular pedicles (two uterine arteries, two utero-ovarian arteries and two utero-ovarian veins, with/without two uterine veins) for uterine revascularization.

We next attempted to determine the best surgical technique for graft revascularization. Numerous animal experiments involving rat, mouse, rabbit, dog, pig, sheep and nonhuman primate models, have used either vascular anastomosis or omentopexy for orthotopic uterine transplantation. In the studies involving omentopexy, all the internal reproductive organs of the animals were removed en bloc; the uterine surface was scraped, and the omentum was wrapped around the uterus and sutured to it to encourage spontaneous revascularization [31–33]. In autotransplanted dogs and macaque monkeys, omentopexy resulted in satisfactory uterine revascularization in more than 70% of animals [32,33]. However, we agree with Dahm-Kähler et al. who questioned whether sufficient uterine blood flow would be re-established before major necrosis would occur in an organ of this size [14]. Thus, neovascularization by omental wrapping does not provide the conditions required for the normal function of the transplanted uterus.

Besides omentopexy, other currently used forms of vascular anastomosis are end-to-end anastomosis between the uterine or hypogastric vessels or inferior vena cavae of the graft and the recipient and end-to-side anastomosis between the uterine vessels of the graft and the external or internal iliac (hypogastric) vessels or inferior vena cava of the recipient As expected, vascular anastomosis was associated with lower rates of necrotic graft degeneration than a nonvascular method. 

Generally, vascular anastomosis is performed in specific ways depending on the vessels to be anastomosed. Two general categories of microvascular anastomosis exist: end-to-side and end-to-end. Although no significant difference in patency or histology has been demonstrated between these techniques, end-to-side anastomosis is preferred by many because of its higher success rate, lower thrombosis rate, and usefulness and technical ease in anastomosing donor and recipient vessels of different diameters [34–36].

In animal models, allogeneic uterine transplantation has been accomplished with end-to-end anastomosis of the uterine arteries and veins [15,17,37] or end-to-side anastomosis of the uterine vessels to the external or internal iliac vessels [12,38,39]. The former procedure can only be applied when hysterectomy is performed in the recipient, and the latter procedure is applicable when the uterus is recovered from a deceased donor [1].

In the present study, with the inclusion of a transplant surgeon to perform vascular anastomosis, we attempted to mimic uterus graft procurement from a live donor. Therefore, we performed end-to-end anastomosis of the uterine arteries and veins. During the retrieval of the uterus, care must be taken to preserve the function of the other organs, especially during separation of the uterine vessels from the ureter and dissection of the distal part of the internal iliac vessels of the graft. In our study, we found small uterine veins deep down in the pelvis, which made their separation difficult; moreover, these veins were too short for subsequent anastomosis, which may have been a factor for the poor outcomes. Therefore, we harvested the uterus together with ovaries and oviducts to take advantage of the ovarian veins, which are much easier to divide and have a much larger diameter, facilitating anastomosis. Moreover, we could use the resumption of ovarian cyclicity (visualized as perineal skin changes) as a non-invasive measure of successful transplantation. Owing to the operative skill of our gynecologic oncology surgeons, uterus graft harvesting was accomplished in approximately 2 h. After 15 min (5–30 min) of the first warm ischemia phase, the donor and recipient vaginal tissues were reapproximated, and vascular anastomosis was performed by the transplant surgeon. Finally, we attached the uterus to the peritoneum on the pelvic side, the native round ligaments and uterosacral ligaments to avoid poor graft fixation [21].

In our study, several methods were used to evaluate whether the uterus was successfully perfused after vascular anastomosis. The most obvious sign of this was that the graft color changed from pale to reddish as soon as the vessels around the sites of vascular anastomoses were unclamped. Moreover, examination of venous effluent samples indicated metabolic stabilization within 180 min after reperfusion, since the pH, lactate and pCO_2_/pO_2_ levels were normalized [14]. 

 The uterus has historically been considered as an immune-privileged organ due to its capacity to carry a semi-allogeneic pregnancy. However, this assumption has been refuted since many animal experiments have proved that a transplanted uterus triggers a normal rejection process, which can only be suppressed by immunosuppressant drugs [40,41]. The use of immunosuppressant drugs during pregnancy and the potential adverse fetal effects have led to much ethical debate. However, in the era of vital organ transplantation, more than 14,000 births have been reported among women with solid organ transplants [42]. The data suggest that there is an increased risk of mild prematurity, decreased birth weight and hypertension/preeclampsia, but no increased rates of congenital malformations were seen [42,43]. Thus, specifically exploring immunological issues relating to uterine transplantation is a necessary part of the inevitable scientific process leading to successful human uterine transplantation. 

The ovine major histocompatibility complex (MHC) has not been fully described [44]. However, it is well-known that the degree of inbreeding between donors and recipients could influence the level of rejection. Inbreeding among farm sheep could reduce the variability of MHC polymorphisms between animals. One-third of sheep from a single farm maintaining excellent breeding practices may be too closely related to mount an immune response against donor tissue [45]. Thus, in the present study, every two sheep were obtained from a single farm. We used an induction protocol including ATG, prednisolone, tacrolimus and MMF, which was similar to the protocols used to decrease the impact of acute rejection after renal, hepatic and cardiac transplantation in humans [46]. Next, standard triple immunosuppression (tacrolimus, prednisolone and MMF) was used as a maintenance protocol before pregnancy was established. In addition, tacrolimus and MMF doses were adjusted according to their blood levels and signs of rejection in order to maintain therapeutic drug levels. Furthermore, MMF was discontinued before pregnancy to avoid fetal malformations [47].

In our study, in spite of the higher dosages than those usually used in humans, the drug AUCs at 12 h were clearly inferior to those recommended for humans, i.e., approximately 5.32 mg/l for tacrolimus. However, we did not adjust the treatment regimen because little is known about immune suppression in ewes. Owing to the digestive specificity of ruminants, along with the rumen volume, pH and rumination, nutrient absorption in sheep differs from that in humans [38]. Thus, a preparatory study to determine the relationship between dose and plasma concentration of immunosuppressive drugs in ruminants should be carried out in the future, or other routes of immunosuppressant drug administration should be used.

 In the present study, we used two methods to assess graft viability. Serial transrectal ultrasonography was carried out from 2 weeks after the uterine transplantation. This showed that both myometrial and endometrial signal intensity were similar to normal, and that there was no necrosis or edema. In addition, ultrasonographic examination of blood flow signals indicated that satisfactory recirculation had been established in the graft; there was no stasis in the anastomosed vessels. One month after the primary surgery, a second-look laparoscopy and laparotomy were performed; some adhesions of the bowel, bladder and omentum, along with a normal-sized uterus and normal-sized ovaries were seen. Gauthier et al. proposed that vaginoscopy could be used to easily explore the cervix [38]; however, in our opinion, observation of the cervix is not sufficient to evaluate the distal part of the graft. Hysteroscopic evaluation, which was recommended by Avison et al., would perhaps be more useful [39,48]. However, even hysteroscopic evaluation is localized to the uterine cavity, and cannot be used to assess the ovaries and uterine myometrium. In addition, the examination is associated with a risk of infection under immune-suppressed conditions. Due to its reliability and excellent imaging quality, Gauthier et al. suggested that pelvic magnetic resonance imaging (MRI) be used to evaluate the transplanted uterus. Considering its ease of operation and abundant availability, we also anticipate that pelvic MRI could offer a means to avoid invasive evaluation methods and serve as a reference examination for graft assessment in the future [38]. In addition, as ICG fluorescence imaging enables real-time observation of uterine hemodynamics [19], this test may be used for the functional evaluation of the transplanted uterus, and will help to determine whether graft necrosis, if present, was caused by mechanical stasis in the anastomosed vessels or by immunological rejection.

Finally, with serial transrectal ultrasonography and a second-look laparoscopy one month later to confirm we successfully performed allogeneic uterine transplantation in sheep. However, the data on embryo transfer or natural conception outcome after the transplantation is still unsatisfactory. Although two of the transplanted uterus showed signs of estrus respectively, with the character of seasonal estrus, ewes didn’t have predetermined estrus time, which brings difficulty for natural conception. In addition, even if the problems of low efficiency and perinatal complications associated with embryo development in vitro appear to be substantively overcome, a number of problems concerning immune regulation and parturition still persist [49], thus embryo transfer for the uterine transplanted sheep is also a complex process. A longer investigation time, more subjects would be necessary. We are currently working to further the study; the findings will be reported in a future article.

## Conclusion

Despite the remarkable progress in the management of infertility in women, there is a notable lack of therapeutic solutions for women with uterine-factor infertility. In the future, human uterine transplantation will become a necessity. Here, we have described a method of orthotopic, allogeneic uterine transplantation in sheep and also described the subsequent immunosuppression protocols. Using these methods, we successfully performed allogeneic uterine transplantation in sheep and made two of the transplanted uterus showed signs of estrus respectively. With the increasing success of experimental studies of uterine transplantation in different animals, we believe that human uterine transplantation will become a reality in the future. Additional studies aimed at the identification of the most suitable combination of immunosuppressive protocols and non-invasive graft evaluation techniques may eventually provide more fascinating targets for effective uterine transplantation.
